# Balint-Ly Obvious: The Value of Balint Groups in Medical Education

**DOI:** 10.1192/bjo.2025.10256

**Published:** 2025-06-20

**Authors:** Roshni Bahri, Ahmad Kamal Mohamad, Sian Davies, Azjad Elmubarak

**Affiliations:** Birmingham and Solihull Mental Health Foundation Trust, Birmingham, United Kingdom

## Abstract

**Aims:** In collaboration with Birmingham Medical School, Birmingham Solihull Mental Health Foundation Trust (BSMHFT) Clinical Teaching Academy piloted a novel Balint Group (BG) scheme for 4th-year medical students during their psychiatry placement. To the best of our knowledge, this is the first time BGs have been accessible to 460 students in one year, and to every student attending psychiatry placement. Research shows that empathy declines in medical students as they progress through their career. However, BGs are known to improve student empathy and support the development of a clinical identity, yet no research has assessed whether through the process of Balint, students successfully gain skills in transference, emotional and cognitive learning, and case mirroring in group dynamics.

**Methods:** All 4th-year students participated in weekly BG sessions during their placement. Upon completing the 4 sessions, students filled out the Balint Group Questionnaire, which assesses three main learning principles: Reflection of Transference Dynamics in the Doctor-Patient Relationship, Emotional and Cognitive Learning, and Case Mirroring in the Group Dynamic. The questionnaire consisted of 15 questions, with a total score of up to 75. The results were analysed using SPSS.

**Results:** 210 students completed the survey. The mean score was 47.1/75. Scores were highest for the Mirroring domain (M=3.42, SD=1.05), followed by Transference (M=3.27, SD=1.04), and Emotional and Cognitive Learning (M=2.94, SD=1.15). A repeated measures ANOVA showed statistically significant differences between the domains. Transference (M=3.27) was rated higher than Emotional and Cognitive Learning (M=2.94), with a mean difference of 0.326 (p<0.001). Similarly, Mirroring (M=3.42) was rated higher than Transference (mean difference –0.152, p=0.022), and Mirroring was also rated significantly higher than Emotional and Cognitive Learning (mean difference –0.479, p <0.001).

**Conclusion:** Higher engagement in Mirroring suggests that BGs help students develop self-awareness and empathy by reflecting on emotional responses to cases, potentially improving patient care and clinical insight. Two more cohorts of students will participate in the study, and we expect similar results with a larger sample size. These findings support the positive role of BGs in medical education.

